# Excluding spontaneous thought periods enhances functional connectivity test–retest reliability and machine learning performance in fMRI

**DOI:** 10.3389/fnins.2025.1730402

**Published:** 2026-01-26

**Authors:** Zhikai Chang, Haifeng Li

**Affiliations:** Faculty of Computing, Harbin Institute of Technology, Harbin, China

**Keywords:** functional connectivity, machine learning, rs-fMRI, spontaneous thought, test–retest reliability

## Abstract

**Introduction:**

Resting-state functional magnetic resonance imaging (rs-fMRI) is a widely used non-invasive technique for investigating brain function and identifying potential disease biomarkers. Compared with task-based fMRI, rs-fMRI is easier to acquire because it does not require explicit task paradigms. However, functional connectivity measures derived from rs-fMRI often exhibit poor reliability, which substantially limits their clinical applicability.

**Methods:**

To address this limitation, we propose a novel method termed time-enhanced functional connectivity, which improves reliability by identifying and removing poor-quality time points from rs-fMRI time series. This approach aims to enhance the quality of functional connectivity estimation without extending scan duration or relying on dataset-specific constraints.

**Results:**

Experimental results demonstrate that the proposed method significantly improves performance in downstream machine learning tasks, such as sex classification. In addition, time-enhanced functional connectivity yields higher test–retest reliability and reveals more pronounced statistical differences between groups compared with conventional functional connectivity measures.

**Discussion:**

These findings suggest that selectively removing low-quality time points provides a practical and effective strategy for improving the reliability and sensitivity of functional connectivity measurements in rs-fMRI, thereby enhancing their potential utility in both neuroscience research and clinical applications.

## 1 Introduction

Functional magnetic resonance imaging (fMRI) has emerged as a widely used non-invasive technology for exploring neurophysiology and identifying biomarkers (Piani et al., 2022). In recent years, there has been an exponential growth in research focusing on resting-state fMRI (rs-fMRI; Buckner et al., 2013). Functional connectivity, which refers to the statistical relationships between the time series of blood-oxygen level dependent (BOLD) signals (Friston, 2011), is a popular method for investigating features of the human brain (Vértes, 2012), making inferences about individual subjects (Gratton et al., 2018), and predicting cognitive behavior (Finn et al., 2015).

Typically, Pearson correlation is commonly used to estimate the functional connectivity matrix, and it has demonstrated relatively high accuracy in identifying individual “fingerprints.” However, it is more susceptible to temporal fluctuations in the BOLD signal compared to other frequency-based connectivity estimation methods (Mahadevan, 2021). Additionally, functional connectivity measurements suffer from poor reliability. Studies have shown that the reliability of functional connectivity can range from poor to moderate (Braun, 2012; Guo et al., 2012; Li et al., 2012), which falls short of clinical standards.

One of the most widely discussed factors affecting the reliability of functional connectivity is excessive head motion, which leads to scan artifacts (Mahadevan, 2021; Van Dijk et al., 2012; Vanderwal et al., 2015; Noble et al., 2019). The reliability of rs-fMRI can be improved by excluding subjects with extreme motion or by regressing out head motion. Furthermore, other factors that may degrade the reliability of functional connectivity include system-related noises (Foerster et al., 2005; Power, 2017), subtle movements during scanning (Power, 2017; Hajnal et al., 1994; Power et al., 2015), and physiological signals such as cardiac and respiratory fluctuations (Evans et al., 2015; Yan, 2009; Chang et al., 2009; Windischberger, 2002; Birn et al., 2006).

Research has shown that functional connectivity with higher test–retest reliability performs better than lower reliability connectivity in machine learning prediction tasks (Guo et al., 2012; Elliott et al., 2019; Wang, 2017). As a result, many researchers have sought to improve the reliability of functional connectivity. For instance Elliott et al. (2019) computed functional connectivity by combining both rs-fMRI and task-based fMRI (t-fMRI) data, Wang (2017) removed volumes associated with strong sleepiness, and Ciric et al. (2018); Gorgolewski (2013); and Zuo et al. (2013) attempted to reduce the impact of motion artifacts.

All these studies aim to enhance the reliability of functional connectivity either by maintaining the length of the time series or by incorporating additional time series data. Even in studies that remove time points related to drowsiness (Wang, 2017), a fixed proportion of time points is discarded, followed by a comparison of reliability metrics between relatively drowsy and relatively alert states. However, the methods mentioned above are challenging to directly apply to other datasets. First, few rs-fMRI datasets include additional t-fMRI data for integration, as used in Elliott et al. (2019). Second, most fMRI datasets lack the necessary physiological signals for regression, and it is also difficult to ensure that the proportion of drowsiness during scanning is consistent across subjects. To address these limitations, we propose an approach to improve the calculation of functional connectivity by removing time points based on a fixed criterion. In this method, we compute the functional connectivity matrix for each subject using a personalized time series length, determined by how many time points are removed according to a consistent threshold. We refer to this new functional connectivity matrix as time-enhanced functional connectivity (TeFC).

We tested our hypothesis on a dataset that includes time-point labels, published by Li (2023), which provides detailed annotations of the periods during fMRI scans when self-generated thoughts occurred. Self-generated thought appears to be an unconstrained mental process that lacks much direction from attention or cognitive control (Mildner and Tamir, 2019). This phenomenon commonly occurs during resting-state fMRI scans and can reduce the reliability of the data (Zuo and Xing, 2014). This process may involve activities such as visual mental imagery, inner language, auditory mental imagery, and somatosensory awareness (Delamillieure et al., 2010; Diaz, 2014), each engaging different brain regions. Consequently, self-generated thought could influence the representation of various brain areas across different networks. Therefore, in our research, we categorized the time points associated with self-generated thought as poor-quality time points in rs-fMRI and removed them from the analysis.

In our study, we used three different types of time series to calculate the functional connectivity matrix for each subject. The first type involved time series after removing the noisy time points, and the resulting matrix was labeled as TeFC. The second type included the entire time series, without excluding any points except for basic preprocessing. The third type consisted of the time points that were dropped, and the matrix calculated from this subset was termed thought functional connectivity (tFC). We then assessed the test–retest reliability of these connectivity measures and used each to train machine learning models. Additionally, we conducted statistical analyses based on the different functional connectivity matrices and compared the results. Ultimately, the TeFC outperformed the original functional connectivity in our experiments, validating our hypothesis that removing poor-quality time points based on a fixed criterion enhances the measurement of functional connectivity.

## 2 Materials and experiments

### 2.1 Datasets

We conducted our experiments on the fMRI data from the Think-Aloud dataset published by Li (2023), which contains 86 healthy adult participants (41 males and 45 females; mean age = 22.1 ± 2.7 years) from the same center. All participants were free from MRI contraindications, psychiatric or neurological disorders, the use of psychotropic medications, and any history of substance or alcohol abuse. As described in Li (2023), each participant was instructed to speak aloud in the scanner whenever self-generated thoughts occurred, with the start and end times of these events being recorded.

### 2.2 Preprocessing

The fMRI data was preprocessed using the DPARSF (Data Processing Assistant for Resting-State fMRI) module within the DPABI (Data Processing & Analysis for Brain Imaging) toolbox (Yan et al., 2016). The preprocessing steps and parameters were as follows:

**Slice-timing correction** (Sladky et al., 2011) to account for differences in acquisition times across slices.

**Realignment** using a six-parameter linear transformation with a two-pass procedure.

**Co-registration** with T1-weighted MPRAGE images.

**Segmentation** was performed using Diffeomorphic Anatomical Registration Through Exponentiated Lie Algebra (DARTEL; Ashburner, 2007).

**Normalization** of the images to the Montreal Neurological Institute (MNI) space using DARTEL, with the voxel size resampled to 3 × 3 × 3 mm.

**Smoothing** with a 4 mm full-width at half-maximum (FWHM) Gaussian kernel.

We did not apply global signal regression (GSR) because there is a great deal of controversy in the application of GSR (Murphy and Fox, 2017; Liu et al., 2017).

### 2.3 Functional connectivity computation

We calculated the functional connectivity using the mean time series extracted from two different templates: the Automated Anatomical Labeling (AAL) template (Tzourio-Mazoyer et al., 2002) and the Schaefer-400 template (Schaefer et al., 2018). The AAL template includes 116 regions of interest (ROIs), while the Schaefer-400 template consists of 400 ROIs. In the Schaefer-400 template, each ROI is assigned to a corresponding network within the seven-network parcellation as defined by Yeo (2011). These networks include the default mode network (DMN), visual, somatomotor, dorsal attention, salience/ventral attention, limbic, and control networks. The time series data for each subject is represented as $X\in {\mathbb{R}}^{R\times T}$ , where R is the number of ROIs, and T is the length of the time series. In this study, R is either 116 or 400, depending on the template used, and T =305.

For each subject, we split the time series into two segments based on the labels of the time points. The first segment is $X_{1}\in {\mathbb{R}}^{R\times T_{1}}$ , which excludes self-generated thought periods, while the second segment is $X_{2}\in {\mathbb{R}}^{R\times T_{2}}$ , consisting only of self-generated thought time points, where $T_{1}+T_{2}=T$ . In this research, the mean $T_{1}$ for all subjects is 178.8 while the mean $T_{2}$ is 126.2. The detailed proportion for each subject can be found in the Supplementary Table S1. We then calculate the Pearson correlation coefficient (PCC) for the two segments $X_{1}$ and $X_{2}$ independently, as well as for the entire original time series X for comparison. The Pearson correlation $\rho_{i,j}$ between the time series of the i -th region and j -th region is computed as follows:


$$\rho_{i,j}=\frac{\mathrm{cov}(x_{i},x_{j})}{\sqrt{\mathrm{var}(x_{i})\cdot \mathrm{var}(x_{j})}}$$

where $x_{i}\in {\mathbb{R}}^{1\times T}$ represents the time series of i -th region. We then estimate the fully connected functional connectivity matrices based on X , $X_{1}$ , and $X_{2}$ , respectively. The corresponding results are $C\in {\mathbb{R}}^{R\times R}$ (functional connectivity), $C_{1}$ (TeFC), and $C_{2}$ (tFC), respectively.

In addition, we also estimate functional connectivity by combining different proportions of $X_{1}$ and $X_{2}$ , and divide $X_{1}$ and $X_{2}$ into four equal segments for subsequent calculations, as will be elaborated in Section 2.4 and Section 2.5.

### 2.4 Gender classification

This dataset’s demographic information, as described in Li (2023), includes data on sex, age, and several psychological scales. Since the dataset consists of healthy young adults, significant differences in these variables are difficult to capture. Therefore, we selected gender classification as the machine learning task. A support vector machine (SVM) is trained for classification. SVM is considered a robust approach for classification and could also be tested as a baseline for performance improvement comparison.

To perform gender classification, we trained SVM models using three different functional connectivity matrices: original functional connectivity, TeFC, and tFC, respectively. For each model, the upper triangle of the functional connectivity matrix was flattened into a feature vector, which served as the input to the SVM. The feature count was 6,670 for the AAL template and 79,800 for the Schaefer-400 template. We evaluated model performance using 10-fold cross-validation, ensuring robust and unbiased results.

Additionally, we trained SVM models based on functional connectivity matrices computed from different proportions of time points $X_{1}$ and $X_{2}$ . We constructed functional connectivity matrices by concatenating varying proportions. For instance, a proportion of 0.4 meant selecting 40% time points of $X_{1}$ and 60% time points of $X_{2}$ , concatenating them to form a new time series, and calculating the functional connectivity from that to train the SVM model. We conducted experiments with proportions ranging from 0 to 1 (0 means the model are trained by $X_{2}$ and vice versa), in increments of 0.05 (i.e., 0, 0.05, 0.1,..., 0.95, 1), for both the AAL and Schaefer-400 templates, which yielded 21 results per template.

### 2.5 Test–retest reliability

To evaluate the test–retest reliability of both TeFC and tFC, we divided the time series $X_{1}$ and $X_{2}$ into four equal segments, treating each segment as a separate session. Each of these segments was independently used to estimate functional connectivity, yielding 16 functional connectivity matrices in total: 4 for TeFC using the AAL template, 4 for tFC using the AAL template, 4 for TeFC using the Schaefer-400 template, 4 using the Schaefer-400 template. To quantify the reliability, we computed the intra-class correlation (ICC) across these different sessions for each connectivity. Specifically, we applied ICC (3.1) as described in Shrout and Fleiss (1979), since we assume that functional connectivity in rs-fMRI should be a stable feature over time. The computation is as follows:


$$\mathrm{ICC}=\frac{MS_{b}-MS_{w}}{MS_{b}+(n-1)MS_{w}}$$

where $MS_{b}$ is the between-subject mean squared strength, $MS_{w}$ is the within-subject mean squared strength, and n is the number of sessions, which in this case is 4. We compute these ICC values using the Pingouin package in Python.

In addition, we also calculated the reliability of several graph-theoretical metrics, such as degree centrality and clustering coefficient. To be more specific, we constructed the undirected graph of functional connectivity based on the threshold from 0.2 to 0.8 and calculate the value of degree centrality and clustering coefficient of each region, respectively. This is designed to discover the reliability of functional connectivity calculation based on different nodes and different strength of connection.

### 2.6 Statistical analysis

To systematically compare different functional connectivity measures at the level of statistical analysis, we applied multivariate distance matrix regression (MDMR; Shehzad, 2014) to identify the primary brain network differences between males and females. MDMR enables parameter-free quantification of whole-brain network reorganization, facilitating unbiased detection of connectomes differences. For our analysis, we filtered the functional connectivity matrices through MDMR to pinpoint key regions exhibiting statistically significant differences (*p* < 0.05) between sexes. We performed the MDMR analysis separately using the original functional connectivity, TeFC and tFC with the Schaefer-400 template, to assess and compare the sensitivity of each functional connectivity measure in capturing sex-based differences at the network level.

We also conducted pairwise t-tests to compare the mean Framewise Displacement (FD) Jenkinson between the two states in order to evaluate the potential influence of head motion on the verbal report. In addition, we compared the DVARS values across the two states to assess differences in BOLD signal fluctuations between conditions.

## 3 Result

### 3.1 Prediction performance

Table 1 and Table 2 show the result of sex classification based on different templates, respectively. On the one hand, in Table 1, the SVM model trained by TeFC has the best accuracy (0.743), recall (0.698), precision (0.826) and AUC score (0.760), while the model trained by tFC has the lowest accuracy (0.552), recall (0.552) and AUC score (0.549). In addition, the model trained by the original functional connectivity and TeFC together with tFC have the moderate performance between TeFC and tFC. On the other hand, Table 2 shows the highest accuracy (0.741) in the model trained by TeFC while lowest (0.649) in tFC. In addition, the model trained by both TeFC and tFC have highest recall (0.762), precision (0.755) and AUC score (0.734). The SVM model trained by tFC have the lowest accuracy (0.649), recall (0.651), precision (0.694) and AUC score (0.655), which is similar to the result of Table 1. Moreover, the model trained by original functional connectivity have the moderate performance between them.

**Table 1:** The performance of SVM model trained by different functional connectivity measures based on AAL template (oFC, original functional connectivity).

Measurement	ACC	REC	PREC	AUC
oFC	0.600 ± 0.155	0.591 ± 0.224	0.753 ± 0.194	0.600 ± 0.176
TeFC	**0.743 ± 0.090**	**0.698 ± 0.196**	**0.826 ± 0.165**	**0.760 ± 0.114**
tFC	0.552 ± 0.172	0.552 ± 0.175	0.710 ± 0.238	0.549 ± 0.218
TeFC+tFC	0.613 ± 0.103	0.598 ± 0.178	0.674 ± 202	0.612 ± 0.111

The values in bold in the table indicate the optimal performance for classification.

**Table 2:** The performance of SVM model trained by different functional connectivity measures based on Schaefer-400 template (oFC, original functional connectivity).

Measurement	ACC	REC	PREC	AUC
oFC	0.718 ± 0.124	0.731 ± 0.201	0.736 ± 0.210	0.715 ± 0.126
TeFC	**0.741 ± 0.136**	0.737 ± 0.280	0.697 ± 0.254	0.725 ± 0.164
tFC	0.649 ± 0.184	0.651 ± 0.233	0.694 ± 0.197	0.655 ± 0.173
TeFC+tFC	0.717 ± 0.124	**0.762 ± 0.225**	**0.755 ± 0.164**	**0.734 ± 0.117**

The values in bold in the table indicate the optimal performance for classification.

Based on the result of Table 1 and Table 2, it can be obviously found that the template of Schaefer 400 is better than the AAL in the area of sex classification. The average performance of it is better than the model trained by AAL template FC even if the worst tFC is used to train the model. In addition, the model trained by both TeFC and tFC based on the Schaefer 400 template may discover more information than only using TeFC.

**Figure 1:**
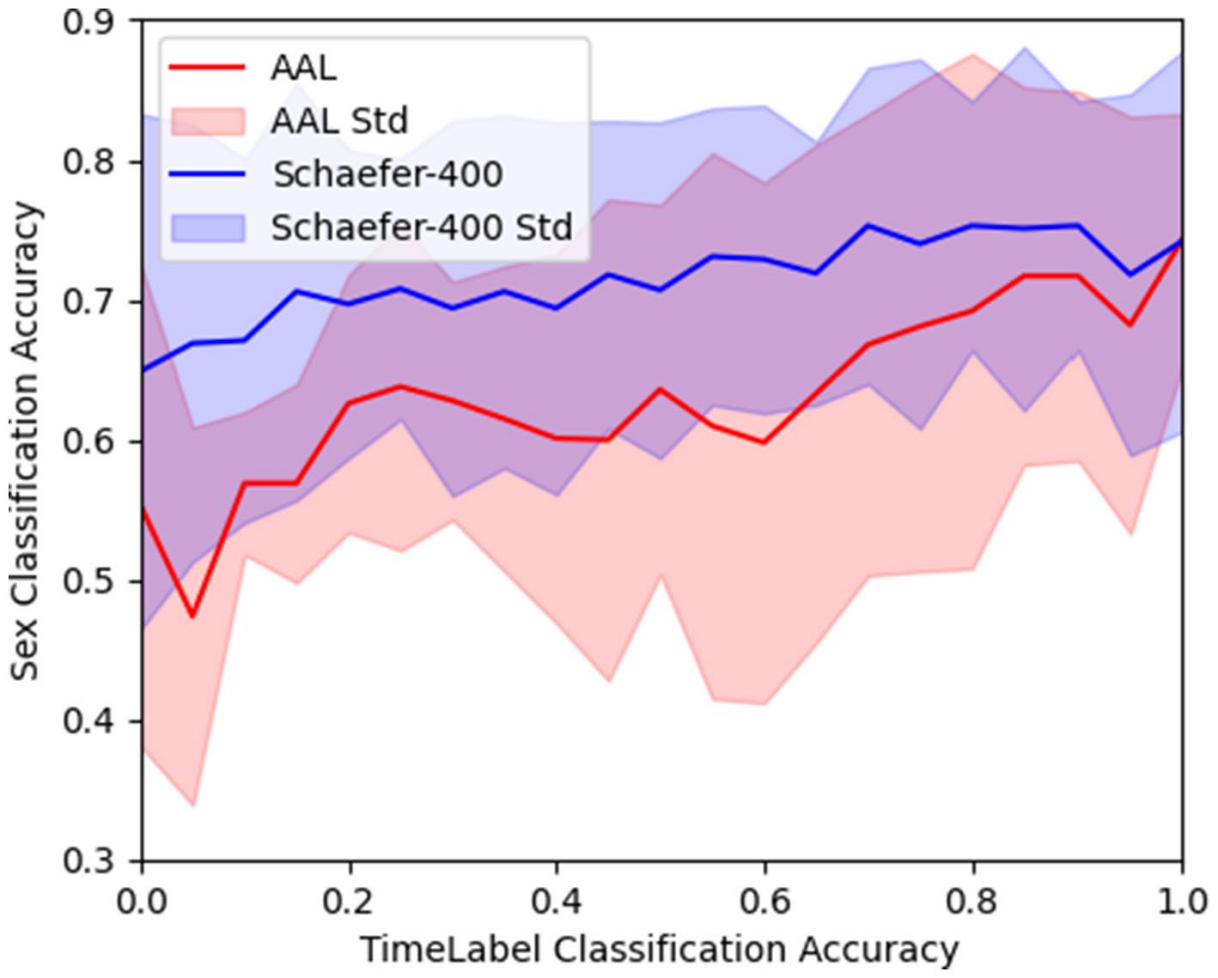
SVM model performance trained on functional connectivity matrices computed with different proportions of the time series.

Furthermore, Figure 1 illustrates the performance of gender classification trained using functional connectivity matrices computed with different ratios of $X_{1}$ and $X_{2}$ . The figure demonstrates that SVM performance in the gender classification task improves as more low-quality time-series data are excluded. Strong associations are observed for both the AAL (Spearman’s R = 0.851, *p* < 0.001) and Schaefer-400 (Spearman’s R = 0.872, p < 0.001) templates, indicating large effect sizes. Drawing from the results in Tables 1, 2, as well as Figure 1, it is evident that the SVM performance remains consistent across templates, with minimal differences observed after most low-quality time points have been excluded.

### 3.2 Reliability analysis

**Figure 2:**
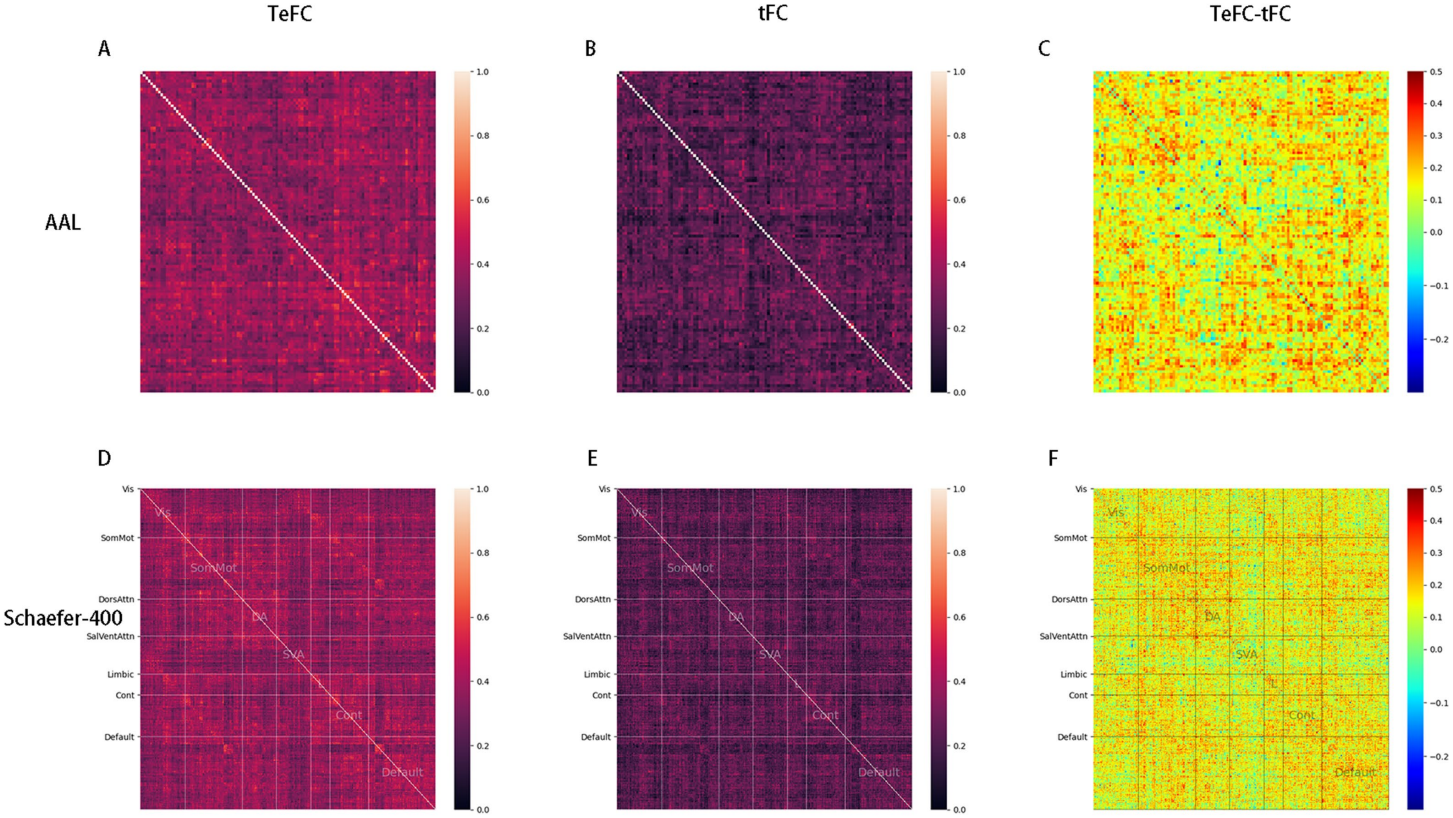
The ICC scores for each value in the functional connectivity matrix. Subfigures **(A,B)** show the ICC scores for TeFC and tFC based on the AAL template, respectively, with subfigure **(C)** illustrating the difference between them. Similarly, subfigures **(D,E)** depict the ICC scores for TeFC and tFC based on the Schaefer-400 template, with subfigure **(F)** showing the difference between them (Vis, visual network; SomMot, somatomotor network; DorsAttn, dorsal attention network; SalVentAttn, salience/ventral attention network; Limbic, limbic network; Cont, control network; Default, default mode network).

Figure 2 presents the test–retest reliability of different functional connectivity computations using Intraclass Correlation Coefficient (ICC) scores. The figure shows that most connectivity values achieve higher ICC scores when calculated with TeFC, regardless of the segmentation template used. Additionally, Tables 3, 4 reinforce this finding, indicating that ICC scores are generally higher for TeFC than for tFC. For the Schaefer-400 template, the highest ICC score for tFC only meets the benchmark for “good” reliability, whereas TeFC achieves this benchmark for 296 connections, with one connection even reaching the “excellent” reliability standard. The average ICC score is also notably higher in TeFC (0.374) compared to tFC (0.234). Similar results were observed in the AAL template. Numerous connections reach the “moderate” reliability benchmark, highlighting the validity of connections between ROIs in resting-state fMRI scans. These results demonstrate that TeFC, which excludes low-quality time points, provides a more reliable and stable measurement of functional connectivity.

**Table 3:** The distribution of ICC scores in AAL template.

ICC category	tFC	TeFC
Excellent>0.75	0	0
Good>0.6	0	11
Moderate>0.4	89	2,332
Poor<0.4	6,581	4,327
Total	6,670	6,670
Mean	0.237905	0.380907

**Table 4:** The distribution of ICC scores in Schaefer-400 template.

ICC category	tFC	TeFC
Excellent>0.75	0	1
Good>0.6	1	296
Moderate>0.4	1723	28,353
Poor<0.4	78,076	51,150
Total	79,800	79,800
Mean	0.23468	0.374409

**Figure 3:**
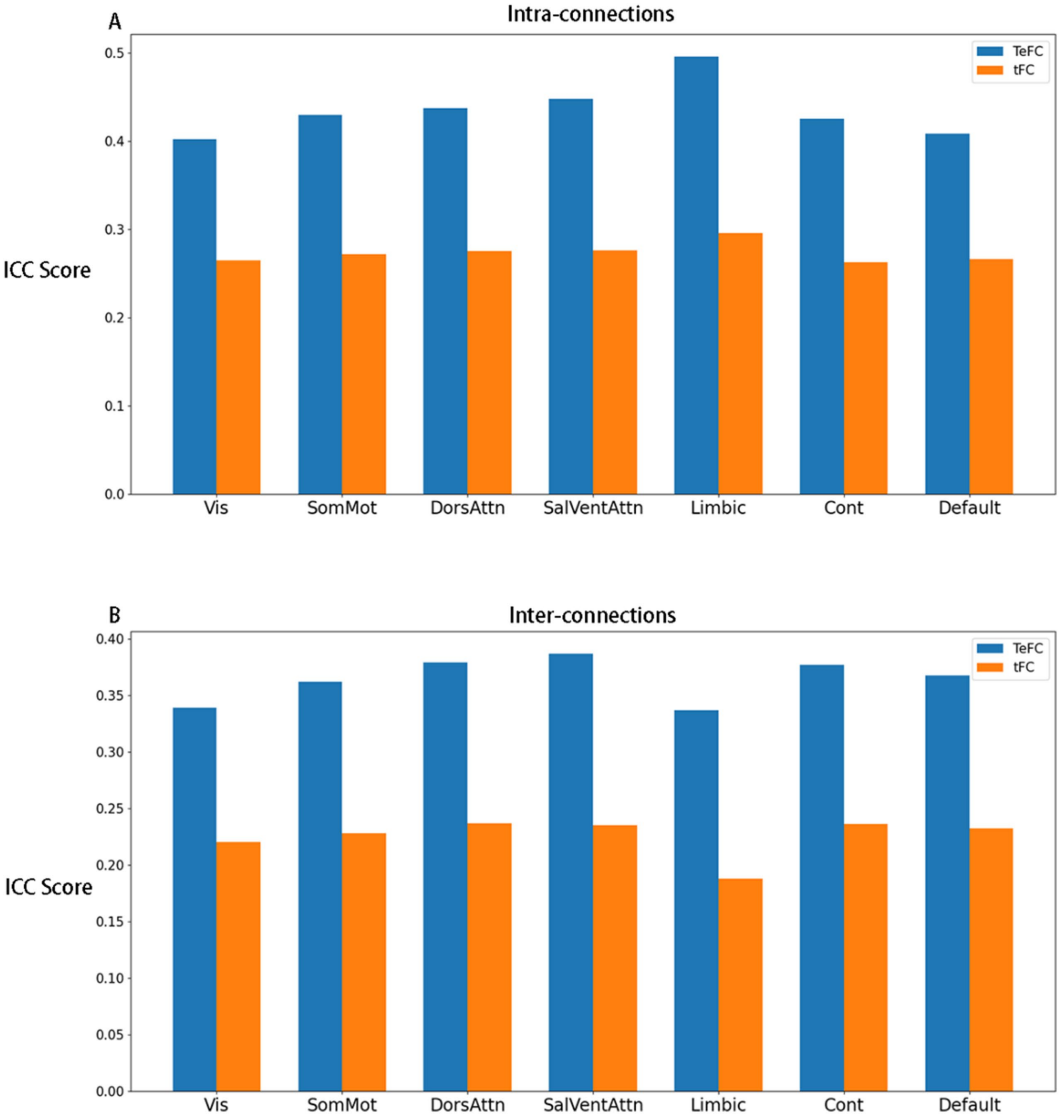
The mean ICC score of each subnetwork, where **(A)** shows the mean ICC score of intra-connections within each subnetwork, and **(B)** shows the mean ICC scores of inter-connections between each subnetwork and other subnetworks. The blue and yellow bars represent TeFC and tFC, respectively (Vis, visual network; SomMot, somatomotor network; DorsAttn, dorsal attention network; SalVentAttn, salience/ventral attention network; Limbic, limbic network; Cont, control network; Default, default mode network).

**Figure 4:**
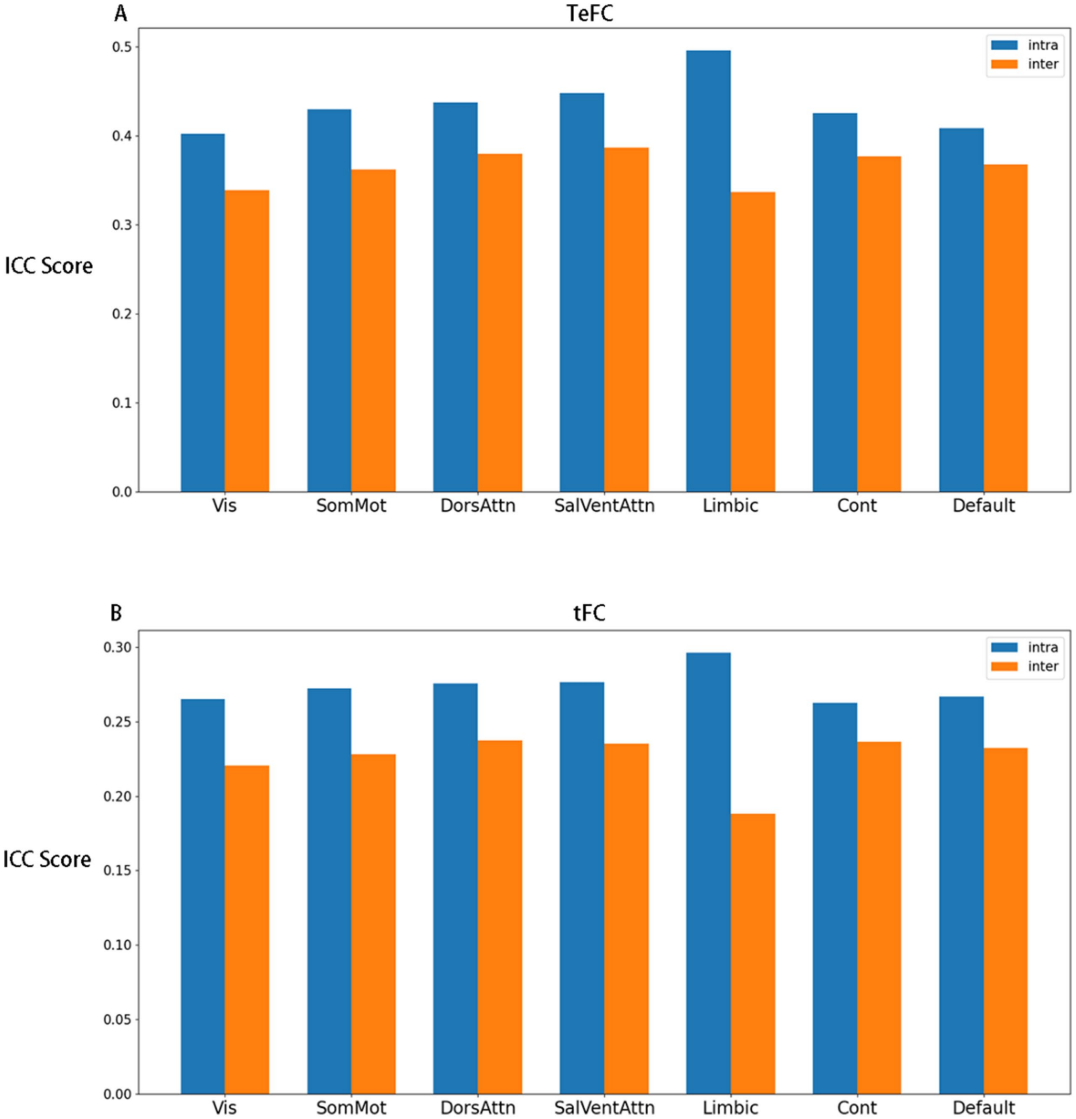
The mean ICC score of each subnetwork, where **(A)** shows the mean ICC score of TeFC and **(B)** shows the mean ICC scores of tFC. The blue bars and yellow bars represent the internal connections of each subnetwork and the external connections with other subnetworks, respectively (Vis, visual network; SomMot, somatomotor network; DorsAttn, dorsal attention network; SalVentAttn, salience/ventral attention network; Limbic, limbic network; Cont, control network; Default, default mode network).

We also observe the ICC distributions in different Yeo subnetworks. In Figure 3, it is evident that all TeFC is more stable than tFC across all subnetworks. Additionally, the limbic network demonstrates the highest average ICC score in intra-connections while exhibiting the lowest in inter-connections, as shown in Figure 4. This phenomenon is present in both TeFC and tFC, and the reasons will be discussed in the next chapter.

**Figure 5:**
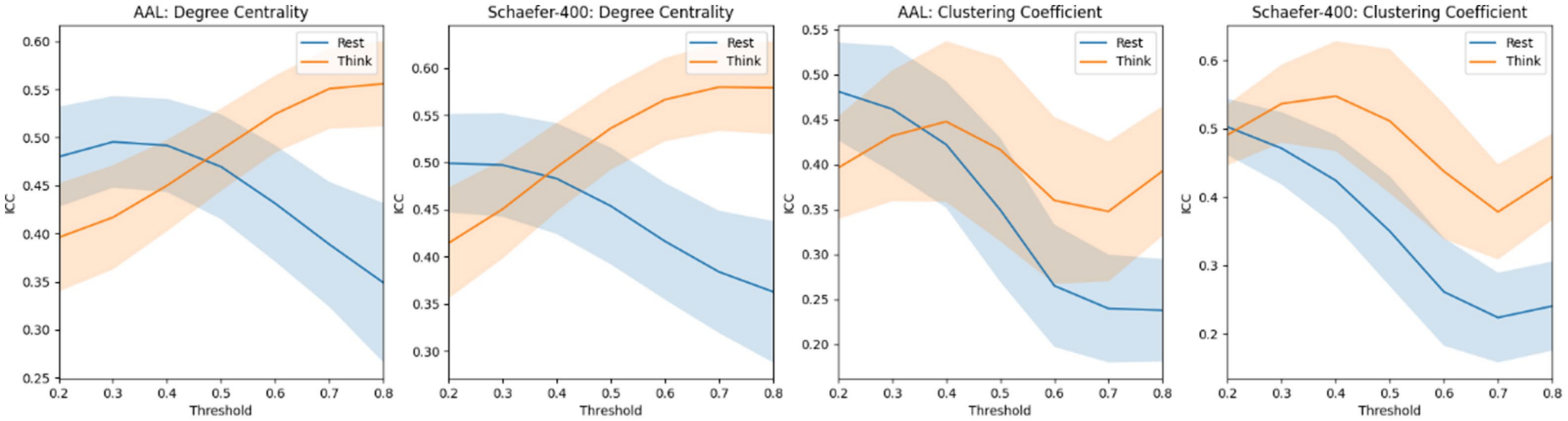
The mean ICC score of degree centrality and clustering coefficient based on the atlas of AAL and Schaefer-400. The blue curve and the yellow curve represent the trends of the mean ICC values of degree centrality and clustering coefficient across all nodes in the resting state and the thinking state, respectively, as the edge connection threshold varies. The shaded areas represent the range of the computed standard deviation.

Figure 5 presents a very interesting phenomenon: when brain networks are computed with lower thresholds, the ICC of graph-theoretical metrics is higher in the non-thinking state; however, when higher thresholds are applied, the ICC of these metrics becomes higher in the thinking state. A consistent trend was observed within each subnetwork. The corresponding results are provided in the Supplementary Figure S1 for details. In addition, we also tried to make the sex classification based on degree centrality and clustering coefficient based on different thresholds. However, it is difficult to achieve stable and accurate gender classification using these metrics; therefore, we did not present the corresponding results in the manuscript.

### 3.3 Data distribution

**Figure 6:**
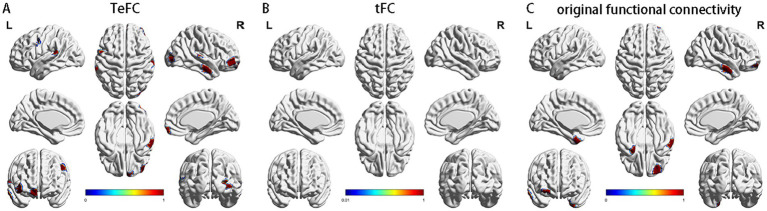
The result of MDMR analysis based on TeFC **(A)**, tFC **(B)**, and original functional connectivity **(C)**, respectively.

Figure 6 demonstrates that Multivariate Distance Matrix Regression (MDMR) analysis using TeFC identifies more significant ROIs than when using original functional connectivity. Specifically, MDMR with TeFC detects 7 significant ROIs (1 area of default mode network in right temporal lobe, 1 area of control network in right lateral prefrontal cortex, 1 area of limbic network in right temporal pole, 1 area of somatomotor network in right hemisphere, 1 area of visual network in right hemisphere, 1 area of salience/ventral attention network in left precentral gyrus, and 1 area of somatomotor network in left hemisphere), whereas the original functional connectivity identifies only 3 (1 area of default mode network in right temporal lobe, 1 area of control network in right lateral prefrontal cortex, and 1 area of limbic network in left temporal pole). Furthermore, MDMR analysis using traditional functional tFC does not reveal any ROIs with significant differences.

### 3.4 Head motion and DVARS

**Figure 7:**
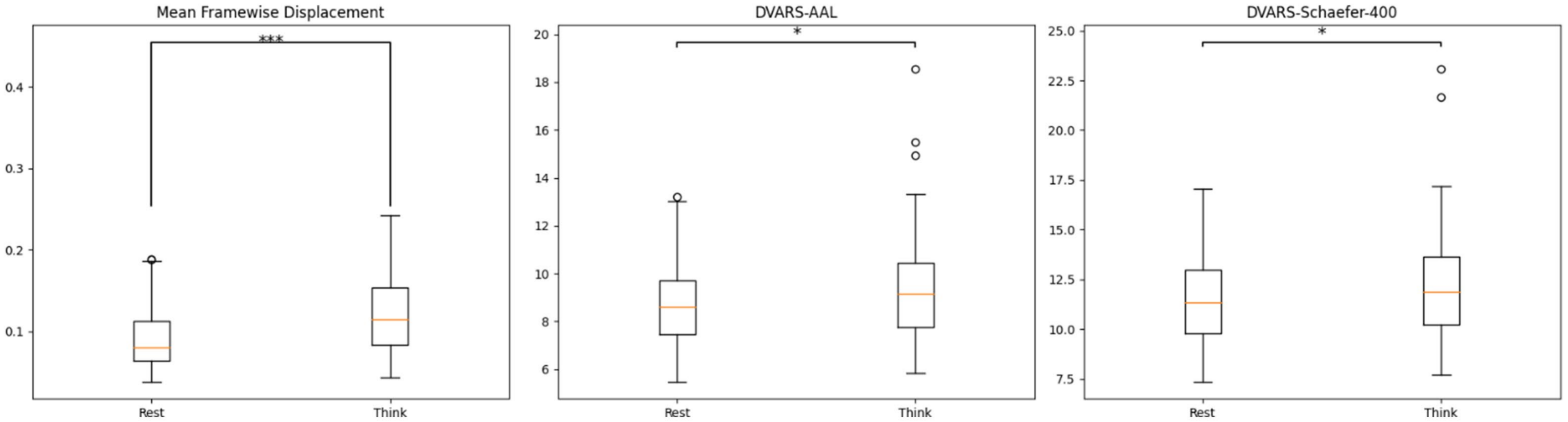
Distributional differences in mean FD (Jenkinson) and DVARS between the “rest” state and the “think” state.

Figure 7 presents the differences in motion- and signal-related quality metrics between the “rest” and “think” states. The mean FD (Jenkinson) is significantly higher in the “think” state compared with the “rest” state (*p* < 0.001), with group-level averages of 0.124 and 0.091, respectively. In addition, DVARS values are also elevated during the “think” state for both the AAL and Schaefer-400 parcellations (*p* < 0.05). Using the AAL template, the mean DVARS values are 8.637 for the “rest” state and 9.353 for the “think” state. Using the Schaefer-400 template, the corresponding values increase from 11.401 (“rest”) to 12.141 (“think”). These results indicate that both head motion and BOLD signal fluctuations are greater during the verbal report period than during rest.

## 4 Discussion

### 4.1 The explanation of results

The results in Table 1 and Table 2 demonstrate the superiority of our TeFC approach in the machine learning task of gender classification, with Figure 1 indicating improved SVM performance as more low-quality time points are excluded. To ensure that the observed results were not driven by insufficient time points, we excluded subjects whose time length in either state was less than 30 time points, as shown in Supplementary Table S1 (3 subjects excluded for the resting state and 12 for the thinking state). SVM models were then trained using the remaining subjects. The results exhibited the same overall trend, indicating that the findings are not attributable to the reduced number of time points. Detailed results are provided in the Supplementary Tables S2, S3.

Moreover, to establish a baseline for gender classification, we trained SVM models using an equivalent number of rs-fMRI scans from the Human Connectome Project (HCP) dataset (Van Essen, 2013). The HCP dataset is a high-quality resource that includes both resting-state and task-based fMRI scans and is widely used across various types of fMRI studies (Elliott et al., 2019; Bedel, 2023; Cho, 2021). In this analysis, we randomly selected 86 non-overlapping resting-state scans using three different random seeds. For each selection, functional connectivity was computed using both the full time series (1,200 time points) and a truncated series containing the first 305 time points, matching the data length used in our study. Each of the six resulting sets of functional connectivity matrices (AAL template) was used to train SVM models for the gender classification task, employing 10-fold cross-validation. The mean classification accuracies obtained from functional connectivity computed using the full time series and the truncated 305-point series were 0.694 and 0.675, respectively. These values are comparable to the accuracy achieved in our study when using the AAL template for sex classification. Furthermore, SVM models trained using all available rs-fMRI scans in the HCP dataset yielded mean accuracies of 0.930 (full time series) and 0.871 (truncated 305-point series). Detailed results are provided in Supplementary Table S4. These findings indicate that the limited sample size in our study substantially constrains the performance of the sex classification models.

Additionally, Table 3 and Table 4 confirm that the remaining time points exhibit higher reliability than those excluded. These findings contrast with the conclusions in Elliott et al. (2019), which propose concatenating time series from different conditions to enhance the reliability of functional connectivity. This discrepancy can be understood in light of Cho (2021), which found that concatenating fewer, more uniform states tends to yield higher reliability. This suggests that data concatenation within a single, stable scan condition—or among more homogeneous and reliable conditions—may better enhance functional connectivity reliability. In contrast, time points associated with self-generated thought are unlikely to meet the criteria for reliable or homogeneous conditions, as these thoughts are unconstrained and lack specific tasks or assignments (Mildner and Tamir, 2019).

In addition, Figure 5 presents a counterintuitive result. The ICC scores of graph-theoretical matrices computed during the resting state are not consistently higher than those computed during the thinking state. Notably, degree centrality and clustering coefficient exhibit higher ICC scores when the brain network graph is constructed using a threshold greater than 0.5. Our explanation is that, in the gender classification task, the model primarily relies on functional connections with relatively low strength. Therefore, the highly reliable strong connections in the thinking state contribute little to capturing effective gender differences. Moreover, directly using these graph-theoretical metrics for classification yields no meaningful results, indicating that the gender classification task does not depend on these metrics.

### 4.2 The cognition load and self-generated thought

According to Li (2023), self-generated thoughts are closely linked to cognitive control, with undemanding environments prompting increased mind-wandering, particularly among individuals with strong cognitive control skills (Kane et al., 2007; Levinson et al., 2012; Smallwood and Schooler, 2015). Consequently, resting-state fMRI scanning, which lacks specific tasks, may lead to extensive mind-wandering and self-generated thoughts. The absence of a structured task guiding participants to focus on consistent content across scans introduces variability in BOLD signal phases, reducing the reliability of resting-state functional connectivity measurements relative to task-based functional connectivity (Greene et al., 2020). While resting-state FC can reveal individual differences that are predictive of task-based performance (Gruskin and Patel, 2022; Elliott et al., 2019) suggests concatenating data from task scans, which maintains phase similarity across individuals. However, it remains uncertain whether the observed benefits result from combining different states or from small, similar segments across scans. Additional studies also highlight the importance of distinguishing between spontaneous brain activities, noting that the BOLD signal time series in resting-state scans are more susceptible to interference from self-generated thoughts in the absence of cognitive engagement, potentially reducing stability and test–retest reliability.

In addition, the self-generated thought segment can be regarded as a task state, in which the only task is “speaking.” As a result, activation within language-related cortical regions is consistently observed (Li, 2023), which may further contribute to a reduction in inter-individual variability (Gratton et al., 2018). Compared with resting-state conditions—where spontaneous thought and unconstrained cognitive processes introduce substantial variability across participants—task states impose structured cognitive demands that synchronize neural activity and attenuate idiosyncratic fluctuations (Cole et al., 2014). This externally driven alignment leads to more homogeneous connectivity patterns, particularly within task-relevant networks, thereby diminishing the extent to which functional connectivity reflects stable, trait-like individual differences. In this sense, task paradigms may enhance the reliability of specific neural circuits but simultaneously constrain the expression of individual variability, whereas resting-state paradigms better capture intrinsic trait-level differences.

However, it is challenging to fully eliminate the impact of self-generated thoughts due to their complexity (Wang et al., 2018), as these thoughts can encompass a range of contents, including images, words, or emotions across multiple dimensions (Gorgolewski et al., 2014). Current technology cannot yet accurately distinguish time points associated with self-generated thoughts; although we attempted to do so, accuracy remained below 70%. As a result, the limitations in accurately identifying and filtering out self-generated thought effects prevent us from directly applying these findings to enhance reliability in other datasets.

### 4.3 The noise during the verbal report period

There are additional physical factors that may influence the test–retest reliability of functional connectivity. As shown in Figure 7, head motion during the verbal report period is significantly higher than during the rest period. Although Li (2023) excluded subjects whose mean FD (Jenkinson) exceeded 0.2 mm in this dataset, several subjects still exhibited mean FD values above this threshold during the verbal report stage. To ensure that these cases did not affect the primary conclusions of the study, we excluded these subjects and repeated both the sex classification and test–retest reliability analyses, obtaining consistent results. Nonetheless, it remains impossible to entirely rule out the possibility that elevated head motion may reduce test–retest reliability, even when the group-level mean FD remains below 0.2 mm. Furthermore, Li (2023) instructed participants to keep their mouths as still as possible during the verbal report period, which likely mitigated head motion to some degree. However, such instructions cannot eliminate the subtle jaw movements relative to the skull that naturally occur during speech, and this component is difficult to remove through standard preprocessing pipelines. Therefore, increased head motion may also be one of the factors contributing to the reduced test–retest reliability observed in this period.

In addition, fluctuations in carbon dioxide (CO₂) may also affect the measurement of BOLD signals. Because the BOLD response reflects a combination of neuronal and vascular contributions (Golestani and Chen, 2020), the relative proportion of these components cannot be precisely determined. Prior work has shown that dynamic CO₂ fluctuations constitute one of the strongest modulators of rs-fMRI signals in gray matter (Golestani et al., 2015). As CO₂ levels typically increase during speech-related behaviors, elevated CO₂ production during the verbal report period represents another potential factor that could reduce the test–retest reliability of functional connectivity.

### 4.4 How to improve the test of rs-fMRI

Studies such as Li et al. (2022) and Raffaelli et al. (2021) have shown that these types of data exhibit similar characteristics even in the absence of overt speech, suggesting that alternative methods may enhance the reliability of resting-state scans. One potential approach is to establish a robust criterion to evaluate the reliability of each time point or interval, allowing us to exclude lower-quality segments and thereby improve functional connectivity calculations. Moreover, we can investigate a deep learning model capable of automatically segmenting the time series into two parts and selecting the more reliable segment for functional connectivity analysis. Other studies have also explored similar techniques; for instance, Jie (2020) proposed using weighted time series to calculate functional connectivity. However, fixed weighting does not account for the variability of real-world conditions, underscoring the need for a personalized segmentation approach to enhance functional connectivity reliability across different individuals.

Additionally, efforts should be made to reduce the proportion of self-generated thoughts and head motion during rs-fMRI scanning. On the one hand, self-generated thoughts can be categorized into intentional and unintentional mind-wandering (Seli et al., 2015; Smallwood and Schooler, 2015), with intentional mind-wandering occurring more often during easy tasks (Martínez-Pérez et al., 2021; Seli et al., 2016). Consequently, the resting-state condition may encourage much intentional mind-wandering (Li, 2023). To address this, participants could be instructed to avoid engaging in self-generated thoughts prior to the resting-state scan, potentially minimizing their occurrence and improving data reliability. On the other hand, we have already observed that the removed time points exhibit more pronounced head motion, suggesting that head motion may contribute to the reduction in test–retest reliability. Although we cannot determine the exact proportion of influence attributable to each factor, we should still make every effort to minimize this source of interference.

### 4.5 Deficiency and future work

In this study, we evaluated the most widely used functional connectivity measure, Pearson correlation. Future research could expand this work by testing other functional connectivity estimation methods, such as Spearman correlation and partial correlation. A key limitation in our study was the availability of suitable datasets; our dataset included only healthy young adults, and other datasets lack time-point annotations. In addition, all data were acquired on 3 Tesla GE MR750 scanners. No other scanner brands were used in this study, which also limits the generalizability of the findings to some extent. This limitation restricts us to conducting only simple classification tasks on a small-scale dataset, and the overall accuracy of gender classification still does not reach the level typically achieved when training on large-scale datasets. In addition, to minimize the impact of speaking on brain activity, Li (2023) also designed a control condition without verbalization, and they obtained consistent brain pattern results. However, in our study, the verbal report still brings some noise to the BOLD signal. However, we cannot use the no verbal report data because there is no specific time label that which time point contains thought, which is also a limitation of this study. The datasets with the label of time points are exceedingly rare. Consequently, we were unable to extend our analysis to pediatric, geriatric, or clinical populations, which often exhibit greater fluctuations during rs-fMRI scans and tend to show lower reliability (Song et al., 2012; Noble et al., 2021; O'Shaughnessy et al., 2008). Additionally, other types of noise—such as fatigue Evans et al. (2015) and Yan (2009) or cardiac fluctuations (Chang et al., 2009; Shmueli, 2007) could also be detected and addressed to improve data quality. This research relied on existing labels for low-quality time points, without implementing a specific paradigm for exclusion. To address this, we are developing a deep learning method that can automatically exclude low-quality time points, which we aim to complete in future work.

## 5 Conclusion

In conclusion, we introduce the concept of TeFC and demonstrate that it is possible to calculate functional connectivity by systematically excluding low-quality time points. The enhancements in reliability and performance in machine learning tasks have been validated, with TeFC showing superior performance in gender classification and exhibiting higher reliability. Future research in rs-fMRI could explore additional criteria for excluding time points, further refining the methodology for analyzing functional connectivity.

## Data Availability

Publicly available datasets were analyzed in this study. This data can be found at: https://rfmri.org/content/rmp-think-aloud-fmri-dataset.
