# Supplementary material for: Excluding spontaneous thought periods enhances functional connectivity test–retest reliability and machine learning performance in fMRI
Source: Front Neurosci. 2026 Jan 26;19:1730402. doi: 10.3389/fnins.2025.1730402 (PMC12883793; doi:10.3389/fnins.2025.1730402)
Supplement: Supplementary file 1 [file Data_Sheet_1.pdf]

Supplementary Material

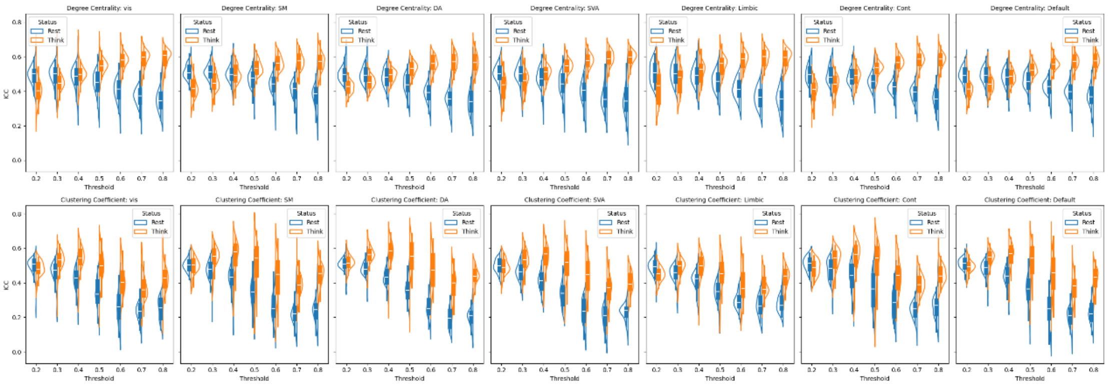

Supplementary Figure 1. Distribution of ICC scores for degree centrality and clustering coefficient in each subnetwork based on the Schaefer-400 atlas. The blue and yellow violins represent the distributions of ICC scores for degree centrality and clustering coefficient across all nodes in the resting state and thinking state, respectively, as the edge connection threshold varies. Abbreviations: Vis = visual network; SomMot = somatomotor network; DorsAttn = dorsal attention network; SalVentAttn = salience/ventral attention network; Limbic = limbic network; Cont = control network; Default = default mode network.

Similar trends to those observed in **Figure 5** and **Supplementary Figure 2** are evident: when brain networks are computed with lower thresholds, the ICC of graph-theoretical metrics is higher in the resting state; however, with higher thresholds, the ICC of these metrics becomes greater in the thinking state. This pattern is consistent across all subnetworks.

Supplementary Table 1. The time length of the two states for each subject.

Subject	Rest (X_1)	Think (X_2)
1	60	245
2	227	78
3	203	102
4	259	46
5	289	16
6	287	18

7	282	23
8	266	39
9	71	234
10	63	242
11	264	41
12	238	67
13	161	144
14	293	12
15	230	75
16	123	182
17	283	22
18	157	148
19	293	12
20	138	167
21	158	147
22	262	43
23	197	108
24	128	177
25	224	81
26	142	163

27	238	67
28	215	90
29	275	30
30	291	14
31	257	48
32	214	91
33	198	107
34	52	253
35	57	248
36	100	205
37	196	109
38	239	66
39	185	120
40	228	77
41	296	9
42	213	92
43	212	93
44	149	156
45	274	31
46	295	10
47	204	101

48	101	204
49	65	240
50	274	31
51	164	141
52	181	124
53	226	79
54	260	45
55	297	8
56	23	282
57	147	158
58	56	249
59	59	246
60	132	173
61	276	29
62	74	231
63	133	172
64	291	14
65	92	213
66	70	235
67	174	131

68	107	198
69	79	226
70	156	149
71	259	46
72	44	261
73	71	234
74	231	74
75	152	153
76	114	191
77	48	257
78	177	128
79	219	86
80	22	283
81	27	278
82	194	111
83	191	114
84	249	56
85	196	109
86	57	248
Mean	178.7674	126.2326

Supplementary Table 1 reports the time length of the two states for each subject. In addition, we trained SVM models after excluding subjects whose time length in either state was less than 30 time points (3 subjects excluded for the resting state and 12 for the thinking state). The performance of the resulting SVM models based on the AAL template and the Schaefer-400 atlas is presented in **Supplementary Table 2** and **Supplementary Table 3**, respectively.

Supplementary Table 2. The performance of SVM model trained by different functional connectivity measures based on AAL template after excluding subjects (oFC = original functional connectivity).

Measurement	ACC	REC	PREC	AUC
oFC	0.621±0.226	0.628±0.321	0.682±0.333	0.657±0.196
TeFC	**0.661±0.184**	**0.650±0.294**	**0.757±0.276**	**0.668±0.156**
tFC	0.509±0.259	0.516±0.288	0.632±0.319	0.514±0.253
TeFC+tFC	0.579±0.187	0.573±0.347	0.527±0.336	0.599±0.212

Supplementary Table 3. The performance of SVM model trained by different functional connectivity measures based on Schaefer-400 template after excluding subjects (oFC = original functional connectivity).

Measurement	ACC	REC	PREC	AUC
oFC	0.704±0.136	0.686±0.299	**0.717±0.282**	0.687±0.152
TeFC	**0.704±0.136**	**0.726±0.306**	0.642±0.274	**0.693±0.143**
tFC	0.648±0.182	0.630±0.303	0.637±0.279	0.608±0.236
TeFC+tFC	0.675±0.158	0.676±0.279	0.712±0.288	0.682±0.134

Supplementary Table 4. The performance of SVM models trained using different numbers of subjects from the HCP dataset.

Scans Number	Time Length	Random Seed	ACC	REC	PREC	AUC
All	1200	-	0.930±0.013	0.944±0.017	0.929±0.018	0.929±0.013
All	305	-	0.871±0.021	0.890±0.024	0.874±0.023	0.870±0.021
86	1200	67	0.667±0.148	0.757±0.162	0.713±0.238	0.707±0.123
86	305	67	0.678±0.125	0.788±0.199	0.707±0.195	0.703±0.132
86	1200	1234	0.697±0.103	0.898±0.137	0.658±0.135	0.678±0.116
86	305	1234	0.733±0.101	0.774±0.169	0.762±0.172	0.740±0.119
86	1200	96	0.719±0.062	0.955±0.094	0.676±0.110	0.690±0.121
86	305	96	0.615±0.127	0.829±0.156	0.621±0.135	0.584±0.164
86	1200	mean	0.694	-	-	-
86	305	mean	0.675	-	-	-

Supplementary Table 4 summarizes the performance of SVM models trained using different numbers of subjects from the HCP dataset. The column "Scan Number" indicates the number of rs-fMRI scans included in the 10-fold cross-validation, while "Time Length" denotes the number of time points used to compute functional connectivity. To perform the 10-fold cross-validation, 86 subjects were selected using three different random seeds, which are listed in the "Random Seed" column. The mean accuracy across all SVM models trained by different random seeds is reported in the final row.
